# “My child before and now”: parental perceptions of changes in functioning following participation in the global integration method (Método de Integração Global) in children with autism spectrum disorder

**DOI:** 10.3389/fpubh.2026.1851875

**Published:** 2026-07-03

**Authors:** Reinaldo da Costa Paulino Netto, Deisiane Oliveira Souto, Iolanda Costa Rodrigues, Amanda Aparecida Alves Cunha Nascimento, Camila de Oliveira Schumann, Millene Cézar Nunes, Ana Clara Schaper Fernandes, Ana Clara de Carvalho Silva, Patricia Aparecida Neves Santana, Thalita Karla Flores Cruz

**Affiliations:** 1 Department of Research and Development, Institute of Neurodevelopment, Cognition and Inclusive Education, Belo Horizonte, Brazil; 2 Department of Occupational Therapy, Postgraduate Program in Occupational Sciences, Federal University of Minas Gerais, Belo Horizonte, Brazil; 3 Department of Psychology, Federal University of Minas Gerais, Belo Horizonte, Brazil; 4 Postgraduate Program in Neuroscience, Federal University of Minas Gerais, Belo Horizonte, Brazil

**Keywords:** autism spectrum disorder, family, functioning, motor activity, psychomotor performance

## Abstract

**Introduction:**

Autism Spectrum Disorder (ASD) impacts children's functioning and participation. Functionality-centered interventions, such as the Global Integration Method (Método de Integração Global—MIG), have been proposed in this context. Understanding parental perceptions is relevant to evaluate their effects on children's daily lives. Objective: To explore parental perceptions of changes in functioning following participation in the MIG program among children with Autism Spectrum Disorder (ASD), interpreted through the framework of the International Classification of Functioning, Disability and Health (ICF).

**Methods:**

This qualitative study was conducted as an embedded component of an ongoing randomized controlled trial evaluating outcomes associated with participation in the MIG program in children with ASD. Parents of children allocated to the intervention group (*n* = 18; aged 6–12 years), diagnosed with ASD and classified at support levels I and II, participated in three post-intervention focus groups.

**Results:**

Findings were organized into three categories: previous experiences with conventional interventions, perceived changes following participation in MIG, and general perceptions of the program. Parents described previous interventions as fragmented, difficult to access, and associated with inconsistent functional experiences. Following participation in MIG, caregivers reported perceived changes related to body functions, particularly motor and communicative aspects, as well as changes in daily activities and social and school participation. Parents also described positive experiences regarding program organization, family support, and expectations regarding future participation and autonomy.

**Conclusion:**

Parents reported perceived changes in functioning following participation in MIG and described positive experiences related to the intervention process.

## 1 Introduction

Autism Spectrum Disorder (ASD) is a neurodevelopmental condition defined by persistent deficits in communication and social interaction, associated with the presence of restricted and repetitive patterns of behavior, interests, or activities (1). Recent epidemiological data demonstrate a continuous increase in its global prevalence, which has intensified the need for therapeutic strategies that go beyond mere symptom attenuation and prioritize the enhancement of functioning, independence, and participation in activities of daily living (2). Current studies indicate that alterations in motor performance, difficulties in sensorimotor integration, and postural deviations are common in children with ASD and are associated with limitations in social engagement, autonomy, and occupational performance (3, 4).

In recent years, therapeutic approaches based on the integration of motor, sensory, and cognitive systems have been increasingly explored in the context of pediatric rehabilitation (5). Evidence suggests that programs combining functional training, postural control, and proprioceptive stimuli promote benefits that extend beyond motor performance, positively impacting attention, behavioral self-regulation, and social interaction (6, 7). In this context, organized movement, when incorporated into structured and functionally relevant settings, acts as a mediator of engagement and learning processes in children with ASD (8).

Within this broader context, the Global Integration Method (Método de Integração Global, MIG) has been proposed as an interdisciplinary intervention approach that integrates motor, proprioceptive, and postural components with the aim of supporting communicative, social, and functional development (5, 9, 10). The program is structured around progressive motor tasks, organized according to a narrative framework and integrated with psychoeducational components, with the aim of reducing sensorimotor unpredictability and promoting action organization. Preliminary findings from an ongoing randomized clinical trial have suggested possible changes in motor and functional outcomes following participation in the MIG program; however, these findings remain insufficient to establish effectiveness and do not explain how such changes may be experienced in daily life.

However, although standardized instruments are essential for measuring therapeutic efficacy, they are not always capable of comprehensively capturing the changes observed in the daily lives of children and their families (11). The literature highlights that parental perception constitutes a valuable source of information regarding functional changes, particularly in the domains of activity and participation, as it reflects the application of skills in natural and ecologically valid contexts (10, 12, 13). Qualitative studies demonstrate that parents are able to identify subtle yet clinically meaningful changes related to autonomy, social interaction, and emotional regulation (14).

The International Classification of Functioning, Disability and Health (ICF) provide a consistent conceptual framework for interpreting these changes by integrating components of body structures and functions, activities, participation, and contextual factors (15). This model expands the concept of health beyond diagnosis and enables the analysis of intervention effects from a biopsychosocial perspective. In the context of ASD, the use of the ICF supports the understanding of the functional impact of interventions by considering not only motor gains but also their implications for participation and daily life (10).

In light of this, it is relevant to investigate how parents perceive functional changes associated with participation in integrated motor interventions in children with ASD and how these experiences are reflected in everyday contexts. Therefore, the present study aimed to explore parental perceptions of the effects of the MIG from the perspective of the International Classification of Functioning, Disability and Health (ICF). By adopting a descriptive qualitative design embedded within a randomized clinical trial, this study aims to complement quantitative outcome measures by exploring dimensions of functioning and participation that may not be fully captured through standardized assessments.

## 2 Methodology

### 2.1 Design and ethics

This qualitative descriptive study was conducted as an embedded component of an ongoing randomized controlled trial (RCT) investigating outcomes associated with participation in the MIG Program in children with ASD (16). The qualitative component was designed independently from the comparative objectives of the RCT and specifically aimed to explore parental perceptions among families whose children received the MIG intervention. Ethical approval was granted by the Research Ethics Committee (approval No. 7,456,658). The parent trial was prospectively registered in the Brazilian Registry of Clinical Trials (RBR-7r6n8zd) and in the WHO Universal Trial Number system (U1111-1326-2272). The study followed the recommendations of the Consolidated Criteria for Reporting Qualitative Research (COREQ) (17). Furthermore, the description of methodological procedures and findings adhered to specific guidelines for qualitative studies in developmental medicine and pediatric neurology (18).

### 2.2 Participants

A purposive sample of parents was selected from a previously established group of families whose children participated in the MIG intervention between 2025 and 2026. Among the children included in the randomized controlled trial (*n* = 66), only families of participants allocated to the MIG intervention arm (*n* = 22) were invited to participate. This purposive sampling approach was selected because the aim of the qualitative study was not to compare intervention groups but to characterize perceived changes and experiences associated with participation in MIG. These children diagnosed with ASD were classified at support levels 1 and 2, were aged between six 6 and twelve 12 years, and had the ability to understand simple commands. Sample adequacy was determined based on thematic saturation. Focus groups were conducted sequentially, and data collection was concluded after completion of the three planned groups, when the research team judged that no substantially new themes or perspectives were emerging from participants' reports and that sufficient depth had been achieved to address the study objectives.

### 2.3 Description of the MIG intervention

The MIG consists of an intensive and interdisciplinary intervention directed at children and adolescents with ASD, based on the assumption that motor, proprioceptive, and postural integration underlies the development of communication, social interaction, and functional performance (5, 9, 10). The MIG group underwent a standardized protocol conducted by physiotherapists, consisting of three 3 weekly sessions of 50 min over five 5 weeks, according to a previously established structure. In all sessions, children wore the MIG Flex therapeutic garment, designed to provide continuous proprioceptive stimulation and postural support during motor tasks. This device contributes to body stabilization and movement organization, while reducing the cognitive demands associated with posture maintenance and motor coordination, thereby facilitating the allocation of attentional resources to interactive, playful, and communicative activities. At the beginning of each session, the physiotherapist adjusted the MIG Flex and initiated a structured narrative, introducing a character, setting, and initial situation, followed by the definition of the central challenge and the explicit identification of the emotional states involved (see Table 1). Additional operational details regarding the intervention structure, standardization procedures, progression criteria, and narrative organization are described in the previously published protocol of the parent randomized controlled trial (16). The problem-solving plan corresponded to the proposed motor activities, integrated with psychoeducational components related to emotions, aiming to promote conceptual generalization and enhance cognitive flexibility through the recognition of common patterns across different symbolic contexts.

**Table 1:** Narrative grammar elements associated with motor activity and the psychoeducational component.

Narrative element	Session content	Motor activity with MIG flex	Integrated psychoeducational component
1. Character and setting	João, a scout, was at a camp with various challenging activities for his emotions. He wakes up early and recalls situations where he struggled to regulate his emotions.	Initial adjustment of the support. The child visualizes the narrative book and the therapeutic environment.	Presentation of a safe context for identifying with the character.
2. Central challenge and emotions	João finds a magical adventure book that teaches him to deal with emotions such as anger, sadness, joy, and fear.	Simulation of the room and boxes where the book is located.	Naming emotions (“It is normal to feel frustrated when something goes wrong”).
3. Resolution plan	To reach the book, João needs to: A) move boxes away (reach); B) lie down to reach further boxes and bring them forward (core).	A) Myofascial mobilization by removing boxes from below and moving them backward. B) Core strengthening by lying down and bringing a box from above the head forward.	Association between persistence (trying again) and motor control (“Let's take a deep breath and try calmly”).
4. Conclusion and generalization	João reaches the book and learns persistence, completing his journey.	Celebratory activity (e.g., jumping or brief dancing with support).	Guided reflection: “How did you feel at the end? Have you experienced something like this at school or home?”

The entire structure of the protocol was contextualized and conducted through a narrative framework, which organized the session stages into movement preparation with active myofascial mobilization, core strengthening, a motor skills circuit (balance, coordination, strength, motor planning, and power), and a contextualized target activity, using equipment such as cones, balls, and unstable surfaces. The program included functional training of motor skills, integrating multiple physical capacities (strength, endurance, balance, flexibility, agility, power, and conditioning) into tasks such as running, jumping, hopping, throwing, and kicking. Sessions were conducted based on the narrative framework and psychoeducational components related to emotions to enhance engagement and the meaningfulness of activities. Tasks progressed gradually in complexity, accompanied by positive feedback and continuous encouragement, and at the end of each session, a home-based task was assigned to reinforce the narrative elements. The motor component of the MIG Program was grounded in the systematic integration of cognitive and behavioral strategies aligned with the principles of predictive coding and embodied cognition, aiming to reduce sensorimotor unpredictability, facilitate action organization, and support the construction of more stable internal models. This included the use of resources such as visual supports, modeling, prompting, task modification, immediate and positive feedback, adjusted instructions, and different practice schedules, with the goal of enhancing engagement, retention, and generalization of motor and functional skills to everyday life contexts.

### 2.4 Data collection

Data collection was conducted through in-person focus groups held after the completion of the MIG intervention, which consisted of 15 sessions. Meetings took place at the Reabilitar Clinic in a quiet environment and lasted approximately 80 min. Parents or caregivers of children allocated to the intervention group of the clinical trial participated in the study. Three focus groups were organized, each comprising six to eight participants. The sessions were moderated by a physiotherapist with experience in pediatric rehabilitation and qualitative methodology, previously trained by the research team and not directly involved in the intervention, in order to ensure impartiality in conducting the discussions. It is noteworthy that there was no prior relationship between the moderator and participants beyond that established during recruitment and informed consent procedures.

Data collection was guided by a semi-structured script developed for the focus groups, aiming to explore parents' perceptions regarding previous interventions received by their children and the effects of the MIG program. The instrument was organized according to the framework of the International Classification of Functioning, Disability and Health (ICF), including questions about changes in motor, physical, and emotional aspects, in the performance and independence of daily activities, and in participation across different contexts and social interactions. Additionally, the script included questions about parents' experiences during the intervention, whether initial expectations were met, the likelihood of recommending the program to other families, and perceptions of long-term impacts, concluding with an open space for additional comments.

Focus groups were conducted immediately after completion of the intervention, consistent with methodological recommendations for focus group studies aiming to explore recent experiences and shared meanings. All sessions were audio-recorded and subsequently fully transcribed for analysis purposes. During the sessions, an assistant researcher observed the discussions and took field notes of an observational nature. Transcripts were returned to participants for verification of accuracy and confirmation of the interpretation of their reports. Each participant took part in only one focus group, and data collection was concluded after the completion of the three planned groups.

### 2.5 Data analysis

The focus group transcripts, integrated with field notes and co-moderator records, were subjected to thematic content analysis conducted in sequential stages (19, 20). Initially, an exhaustive reading of the material was carried out to ensure familiarization with the data. Initial codes were generated inductively from recurrent units of meaning identified across transcripts and field notes in relation to the study objectives. Subsequently, two researchers with different backgrounds and experience in pediatric rehabilitation independently segmented the text into units of meaning and performed coding. After independent coding, the researchers compared and discussed the identified codes, grouping them into broader thematic patterns through an iterative analytical process. The results were then systematized for interpretation and synthesis of findings. Agreement between coders was verified through investigator triangulation, and any discrepancies were discussed until consensus was reached, with the involvement of a third researcher experienced in qualitative research when necessary. Themes and categories were progressively refined through repeated examination of the material and organized into categories named according to the term that best represented their content. After completion of the thematic analysis, the ICF framework was used as an interpretative and organizational model to support the presentation of findings according to domains of functioning.

## 3 Results

Of the 21 parents of children in the MIG intervention group, three were unable to attend the focus group sessions due to personal reasons. Thus, eighteen 18 parents (85.71%) participated and were organized into three focus groups of six parents each. The characteristics of the children are described in Table 2. The mean adherence of the children to the MIG program was 91.5%. The results were grouped into three categories: (I) perceptions of previous conventional treatments, (II) benefits of MIG across the ICF components of functioning, and (III) general perceptions of MIG. The number of parents who provided input in each subcategory varied. Approximately 72.2% (*n* = 13) of parents reported experiences with previous conventional treatments. Regarding the functionality category, 94.4% (*n* = 17) provided reports related to body structures and functions, 83.3% (*n* = 15) to the activity domain, and 88.9% (*n* = 16) to the participation domain. All parents (100%; *n* = 18) provided reports regarding their perceptions of the MIG program.

**Table 2:** Characteristics of the children.

Variable	Value
Sample size	18
Mean age of children (SD)	11 years (2.42)
Sex of children, *n* (%)	
Male	15 (83.33)
Female	3 (16.7)
Support level, *n* (%)	
Level 1	12 (66.7)
Level 2	6 (33.3)
Relationship of respondent, *n* (%)	
Mother	16 (88.9%)
Father	2 (11.1%)

### 3.1 Perception of previous conventional treatments

The results of this study showed that parents described previously accessed conventional treatments as heterogeneous experiences, marked by both specific benefits and structural and therapeutic limitations. Some participants reported difficulties in accessing services, especially within the public healthcare system, including long waiting lists, early interruptions, and discharge due to service overload, which compromised continuity of care. The most frequently mentioned interventions included psychology, speech therapy, and occupational therapy, delivered through the public health system or private services. Access to the private sector was associated with better continuity and conditions of care, but was limited by financial constraints.

Regarding perceived effectiveness, some parents acknowledged progress in the development of communication, motor coordination, and the diagnostic process, particularly when there was a stronger bond with the professional and continuity of care. In contrast, others reported little or no functional change, as well as dissatisfaction with the superficial nature of the interventions and the lack of guidance for families, which hindered the generalization of gains to the home environment. Thus, parental perception points to a fragmented care scenario, in which outcomes depend heavily on treatment regularity, the quality of the therapeutic relationship, and the possibility of integration between services and the family. Some parental reports are presented in Box 1.

Box 1 **Parents' reports on previously conducted conventional treatments.**

• “I noticed that it didn't make much difference—to be honest, he would leave therapy the same as when he arrived.”
• “It was positive because occupational therapy worked quite well—he developed a lot—and in speech therapy he was able to communicate better.”
• “He regressed; my child was supposed to move forward, but he was going backward.”
• “It wasn't a treatment as appropriate as it should have been.”

### 3.2 Parental perceptions of changes following participation in the MIG program across ICF components of functioning

#### 3.2.1 Body structures and functions

Parental perceptions indicated changes in different aspects of the body structures and functions domain, with emphasis on neuromusculoskeletal and movement-related functions. Reports described changes in muscle strength, balance, postural control, and both gross and fine motor coordination. Parents noted greater firmness in hand grip, better organization of body alignment in sitting and standing positions, increased trunk stability, and changes in lower limb movement patterns. Modifications in overall motor control and coordination between body segments were also mentioned, as well as improvements in the use of upper limbs for object manipulation. Some caregivers identified more evident advances in gross motor functions compared to fine motor functions, indicating non-uniform progress across components of the neuromusculoskeletal system (see reports in Box 2).

Box 2 **Reports of the impact of the MIG program on body structures and functions.**

• “He has improved about 50% in hand stability and also in balance.”
• “His posture has improved a lot. Before, he was very hunched over; now he can sit properly and adjust himself, he even notices when he is sitting incorrectly.”
• “I noticed that he is no longer walking on his tiptoes; now he places the sole of his foot firmly on the ground.”
• “Before, she didn't say anything or ask for anything. Nowadays, she knows how to ask, she knows how to speak, even the time, she can tell.”
• “Now I think that when she grows up, she'll be ready to work, because… I should have recorded how she was before, she didn't even talk to us. Everything was by pointing; now you can say anything to her and she answers you. Wow, you should see it! I'm amazed at how much she has changed!”

Regarding speech, cognitive, and behavioral functions, reports indicated changes in expressive language, communicative responsiveness, and the expression of emotional states. Parents described increased verbal output, greater clarity in speech, and an improved ability to express feelings. Changes were also noted in mental functions, including attention, behavioral organization, and control of restlessness, as well as aspects related to emotional regulation. These aspects were described in comparison to the period prior to the interventions, forming a set of descriptions centered on body functions, particularly within the motor, communicative, and mental systems, without direct reference to the activity or participation domains.

#### 3.2.2 Activity

Parents reported changes in their children's daily activities across different domains. In the mobility component, behaviors such as beginning to run, experiencing less fatigue during walking, going up and down stairs with less fear, walking with greater confidence, climbing onto objects with more stability, and moving around with fewer falls were described. Regarding activities of daily living, caregivers reported attempts and partial performance of tasks such as using a fork and knife, holding a cup without spilling, dressing with less assistance, choosing their own clothes, putting away clothes and objects, carrying grocery bags, brushing teeth, and combing hair with supervision. Changes were also mentioned in simple household tasks, such as helping to put away groceries and organizing personal belongings. Box 3 presents some reports provided by parents during the focus groups.

Box 3 **Parents' reports on the benefits of the MIG program in the activity domain.**

• “Before, he couldn't run… now he runs. He didn't have the confidence to go up the stairs, and now he goes up and down.”
• “He didn't know how to tie his shoelaces. Before, every day he would call me: ‘Mom, come tie it with me.' Now he can tie his shoelaces by himself.”
• “He is now able to eat better without spilling and holds the cup more accurately.”
• “Now he says, ‘Mom, I got hurt and I fell because I was running.”'
• “She can now go up and down the stairs on her own. Before, she needed help; now she does it independently. After that, she also started playing more, drawing, and coloring, things she didn't do before.”
• “He is more cautious now and no longer interrupts people all the time.”

Parents also reported changes in activities related to functional communication in daily life, such as speaking more, responding when called, describing events from their day, requesting objects, and greeting others. Regarding play and manual activities, increases were described in time spent in independent play, greater initiative to draw, color, assemble toys, and perform manual tasks, as well as greater involvement in games that require body movement. Reports of difficulty remained in activities requiring fine motor coordination and in situations with excessive auditory stimuli, such as supermarkets and parties, although changes were noted in tasks performed in daily life.

#### 3.2.3 Participation

Parental reports indicated changes related to the participation component, particularly regarding children's involvement in school and community contexts. Parents described that, after the intervention period, children were present more regularly in collective situations, such as school activities, interactions with other children, and social events, compared to the period prior to the intervention. These changes were associated with greater persistence in these environments and a higher willingness to engage in routines outside the home.

In the domain of interpersonal relationships, caregivers reported an expansion in contact with peers and family members, with greater engagement in social interactions and increased acceptance of shared environments. Changes were also noted in how children participated in public situations, including greater tolerance of the presence of others and increased involvement in group activities. Overall, the reports indicated that children began to more frequently occupy social and community spaces, reflecting transformations in the participation component. Box 4 presents some parental reports regarding the improvements they observed in the Participation domain.

Box 4 **Parents' reports on improvements in participation.**

• “Now he is able to stay longer at school. Before, when I went to pick him up, he already wanted to leave and didn't really participate. Today he stays, takes part in the activities, and no longer asks to leave early like before.”
• “Before, he didn't like going to parties, he would stay more isolated, in a corner. Now he goes, stays among the other children, and participates more. He doesn't withdraw like he used to; he stays with the others.”
• “Now he accepts going out, walking in the street, and going places with us. Before, it was only at home, he didn't want to go out. Today he joins us more and stays in environments outside the home.”
• “He started playing more with his cousins and other children. Before, he stayed more by himself and didn't get involved much. Now he seeks out interaction and spends more time playing and engaging.”
• “My son didn't approach anyone before. Even our friends used to say, ‘your son doesn't talk, right? He seems like a wild child,' without understanding the issue. He has improved so much.”

### 3.3 General perceptions of the MIG program

The experiences reported by parents regarding the MIG program primarily emphasized how the interventions were conducted and experienced throughout the participation period. Caregivers described the program as organized, welcoming, and tailored to the children's needs, highlighting the combination of play-based, physical, and communicative activities as a distinguishing feature of the approach.

Regarding expectations and overall evaluation, parents reported that the observed outcomes exceeded their initial expectations, even considering the short duration of the intervention. Their statements indicated surprise at the changes observed and at the children's engagement in the proposed activities, as well as recognition of the program's potential long-term impact.

Caregivers associated the program's effects with the possibility of greater future autonomy, better preparedness for social situations, and increased self-confidence in the children. Recommendations of the program to other families were frequent, justified both by the perceived benefits and by the lack of accessible, high-quality services.

**Figure 1:**
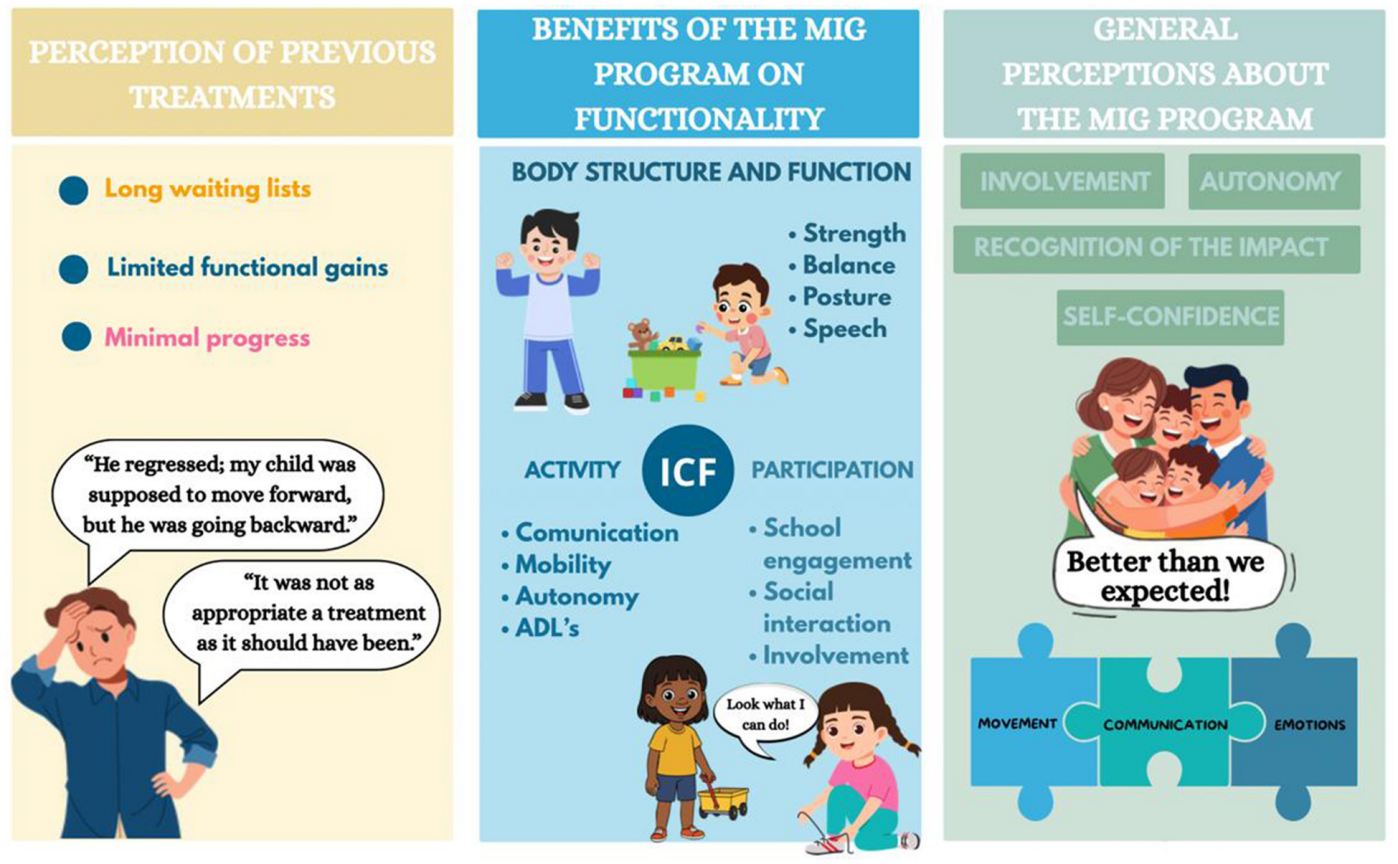
Summary of the main findings of this study.

Additionally, parents highlighted the relevance of the program as a space for support, exchange, and guidance, helping to reduce feelings of insecurity and isolation previously experienced. Overall, the experience with the MIG program was described as transformative, not only for the children but also for the caregivers themselves, who reported feeling more supported and confident in managing the demands related to their children's development. Some participant reports are presented in Box 5. Figure 1 summarizes the results of this study.

Box 5 **Parents' reports on their perception of the MIG program.**

• “It exceeded expectations, right? Because I thought maybe he wouldn't be able to achieve that much. I didn't expect him to improve this way, especially in confidence when walking and holding things.”
• “For me, it exceeded my expectations because I didn't think he would achieve even half of what he did. In less than two 2 months, I look at my son today and see a different person.”
• “At first, I was a bit skeptical, but it exceeded my expectations. It was a great experience. He asks every day if he has therapy.”
• “It was extremely rewarding. Seeing my son's progress each week was amazing. It's like a step-by-step process, every single day.”
• “I see progress in my son, I see a future. With each passing day, Vinícius is improving.”

## 4 Discussion

The present study aimed to investigate parental perceptions of the effects of the MIG program from the perspective of the ICF. The research involved 18 parents of children with ASD who participated in a clinical trial that evaluated the impacts of the MIG program. The qualitative analysis of the reports revealed that parents identified significant benefits across multiple components of functioning, particularly in body structures and functions, including muscle strength, balance, gross and fine motor coordination, posture, and communication skills, as well as improvements in attention, emotional regulation, and behavior. Additionally, caregivers observed changes in the children's activities and participation, with greater engagement in activities of daily living, play, manual tasks, and school and community contexts, demonstrating perceived effects even after a relatively short intervention period. All participants reported satisfaction with the intervention, highlighting perceived changes in the children's daily lives and the support provided to caregivers, suggesting that families perceived changes in functioning and wellbeing following participation in the program.

In this study, parental reports on previous conventional treatments, described as heterogeneous, fragmented, and with limited functional outcomes, reflect problems widely documented in the literature on services for children with ASD. Evidence indicates that, although international guidelines for early and continuous interventions exist, their implementation in the public sector is often hindered by structural barriers, such as a shortage of specialized professionals, long waiting times, and discontinuity of care, factors that reduce the effectiveness of interventions in clinical practice (21, 22). These obstacles are consistent with studies that associate greater therapeutic continuity and stronger professional relationships with better outcomes in the development of communication and motor skills (23). In contrast, intermittent or superficial therapies tend to limit the generalization of gains to natural contexts, which aligns with reports of “little or no functional change” and dissatisfaction with family guidance. The literature also indicates that active family involvement and the integration between clinical services and the home environment are key determinants for transferring therapeutic gains to the daily life of children with ASD (10, 24). Thus, the findings of this study reinforce that, in the absence of continuous, integrated, and function-oriented care models, the perceived effectiveness of conventional treatments tends to remain limited, regardless of the type of formal intervention adopted.

Parental reports described perceived changes in body structures and functions following participation in the MIG program, particularly in strength, balance, posture, and both fine and gross motor coordination, as well as in communication skills and emotional regulation. These findings are consistent with studies showing that motor interventions and physical activity programs can improve motor skills, postural control, and object manipulation in children with ASD (25, 26). Moreover, evidence suggests that motor development may positively influence communication and behavior, supporting parents' perceptions of greater clarity in speech and expression of feelings (27, 28). Thus, the results reinforce the importance of integrated approaches that simultaneously address motor, communicative, and cognitive-behavioral systems in children with ASD.

Parental reports of improvements in strength, posture, coordination, and motor control reflect the importance of interventions that promote neuromusculoskeletal stimulation in children with ASD. Changes in gait, grip strength, and postural alignment suggest that structured programs can modulate motor development, reinforcing evidence that motor deficits in ASD are associated with difficulties in sensorimotor integration and postural regulation (29). The differentiation of gains between gross and fine motor skills observed by parents also corroborates studies indicating uneven progress across these domains, highlighting the need for specific strategies tailored to each type of skill (30). Furthermore, the perceived improvement in expressive communication and emotional regulation points to interrelationships among motor, cognitive, and social systems, which is consistent with findings suggesting that interventions integrating movement, attention, and expression may promote overall development in children with ASD (31). These results reinforce that a focus on body structures and functions, when applied systematically, can generate broad benefits that extend beyond the purely motor domain, impacting children's functional interaction in daily life.

Reports of changes in daily activities suggest that parents perceived greater functional autonomy following participation in the intervention, particularly in tasks that require motor planning, coordination, and self-care. The increased ability to perform activities of daily living, such as dressing or handling utensils, supports studies suggesting that combined programs of motor stimulation and functional practice can reduce caregiver dependence and improve adaptive competence (32). Improvements in mobility and participation in independent play were also observed and may be associated with greater motor confidence, which enhances opportunities for learning and environmental exploration (29). Remaining difficulties in fine motor tasks and in contexts involving sensory overload reflect patterns described in the literature, indicating that children with ASD show significant variability in fine motor skills and sensory sensitivity, requiring specific intervention strategies (33). These findings reinforce the importance of programs that integrate functional, play-based, and motor activities, aiming not only for isolated gains but also for the generalization of skills to the child's daily life.

Parental reports indicate that, following the MIG program intervention, children began to show greater presence in school, social, and community contexts, demonstrating an expansion in participation, one of the most affected domains in children with ASD. Caregivers reported increased time engaged in school activities, greater involvement in peer play, and a higher willingness to attend environments outside the home, suggesting that MIG enhanced opportunities for social engagement that were previously limited. These changes may be related to improvements in socioemotional and self-regulation skills, such as increased tolerance to external stimuli and a greater ability to wait their turn and follow group rules, factors that are often limiting in this population (34, 35). Reports of greater initiative in interactions with cousins, peers, and family members indicate advances in functional communication and social confidence, with potential impacts on autonomy and quality of life (36). These findings reinforce that structured and multimodal programs, such as MIG, which integrate motor, play-based, and interactional activities, can contribute to reducing social isolation and promoting gradual participation in natural environments. However, the literature indicates that, although these gains are noticeable, they often require ongoing support in more complex sensory and social contexts, highlighting the need for individualized follow-up and integration of interventions into family and school routines (37).

The findings indicate that families' experiences with the MIG program were evaluated positively, both in terms of the perceived gains in children and the way the intervention was organized and conducted. The literature highlights that qualitative studies in pediatric rehabilitation are essential for understanding how families interpret the effects of interventions and how these effects translate into expectations of greater autonomy and preparedness for social situations, factors associated with children's engagement and adherence to treatment (38). Parents' surprise and the surpassing of initial expectations follow a pattern described in the literature, in which observable changes in motor performance and social initiative are quickly recognized as signs of overall developmental progress (38). Furthermore, the appreciation of the MIG program as a space for support, exchange, and guidance is consistent with evidence that family-centered care programs reduce feelings of insecurity and isolation while strengthening parental competence and hope regarding the child's future (39). Thus, the overall positive evaluation of the MIG program, combined with frequent recommendations and expectations of lasting effects, reinforces that integrated motor interventions, when accessible and tailored to individual needs, produce impacts that extend beyond the motor domain, reaching emotional, social, and family dimensions of development.

The limitations of this study include, first, the small sample size and the use of a purposive sample composed only of parents of children from the MIG group, which limits the generalizability of the findings. The exclusive inclusion of children with ASD at support levels 1 and 2 restricts extrapolation to cases with greater support needs. The data are based solely on parental reports and are therefore subject to expectation, gratitude, and social desirability biases. Data collection took place immediately after the end of the intervention, preventing analysis of the maintenance of effects in the medium and long term. Additionally, the focus group dynamics may have favored the predominance of positive perceptions. Finally, it was not possible to fully control for the influence of other concurrent interventions or changes in the family and school context, which may have contributed to the perceived outcomes.

## 5 Conclusion

In summary, this study explored parental perceptions of changes in functioning following participation in the MIG program, including body structures and functions, activities, and participation in children with ASD. Reports indicated improvements in motor skills, functional communication, performance in daily activities, and greater inclusion in social, school, and community contexts. Additionally, parents highlighted the organization, the play-based nature, and the program's adaptation to individual needs, as well as its role as a space for support and family guidance. These findings contribute to understanding how families interpret changes experienced in daily life following participation in integrated motor interventions to promote functional gains perceived in daily life and to expand opportunities for participation, while also emphasizing the relevance of qualitative approaches for understanding the meaning of these changes in families' lives. Future studies should investigate the sustainability of these effects and their relationship with objective measures of functioning in larger and more diverse samples.

## Data Availability

The raw data supporting the conclusions of this article will be made available by the authors, without undue reservation.
